# Risk, Resource, Redemption? The Parenting and Custodial Experiences of Young Offender Fathers

**DOI:** 10.1017/S1474746415000500

**Published:** 2016-01

**Authors:** Linzi Ladlow, Bren Neale

**Affiliations:** * School of Sociology and Social Policy, University of Leeds E-mail: l.ladlow@leeds.ac.uk; ** School of Sociology and Social Policy, University of Leeds E-mail: b.neale@leeds.ac.uk

**Keywords:** Young fathers, young offenders, parenting programmes, risk, redemption, desistance, life course perspectives

## Abstract

Drawing on an ESRC funded qualitative longitudinal study of young fatherhood, this article explores the experiences of young offender fathers, the complex intersection of offender and fatherhood pathways for young men and the impact of professional support and tailored intervention programmes on these processes. The article challenges the axiom of young offender fathers as inherently ‘risky’, and suggests the utility of a dynamic, life course approach to criminal policy and practice that recognises the fluidity of their life journeys, and brings ideas of redemption more centrally into the picture.

## Introduction

Disadvantaged young fathers (defined here as those under the age of twenty-five at entry into parenthood) are likely to face a raft of challenges in assuming a parenting role and identity (Neale, forthcoming). For those who have spent time in the Criminal Justice System (CJS), these problems may be magnified. They are more likely to come from disadvantaged backgrounds, facing challenges associated with poverty, lack of social support, volatile relationships, mental health problems and low educational attainment (Buston *et al.*, 2012; Neale and Davies, forthcoming). Maintaining contact with their children and fulfilling a role as a parent in the secure estate is a particular challenge (Meek, 2007b). Upon resettlement, having a criminal record may compound the problems of finding employment and securing housing appropriate for children. This in turn may affect their credentials and contributions as fathers, exacerbating their popularly perceived ‘feckless’ identity, and locating them, seemingly indelibly, on the margins of mainstream society.

More broadly, youths in custody are among the most vulnerable in society. By the time they find their way into the CJS, most will have already received some form of professional intervention, with 71 per cent having been involved with, or in the care of, social services (Youth Justice Board, 2007). Official statistics on the fatherhood status of incarcerated youths are not routinely collected (Meek, 2011), but estimates suggest that young offenders are six times more likely to be fathers than non-offenders of the same age (Dennison and Lyon, 2001), and that between 25 per cent and 50 per cent of young offenders are fathers or expectant fathers (Meek, 2011; Buston *et al.*, 2012).^1^

### The risk framework

It is commonly assumed that young men are irresponsible and likely to engage in risky behaviour. This view is reinforced for young fathers, who are perceived as a risk to, rather than a resource for, their children (Featherstone, 2013). An often-cited risk factor of future criminality is having a parent who has been in prison (Dallaire, 2007), an assumption of an intergenerational cycle of offending that can further label these young men. Young offender fathers, then, are perceived to have multiple ‘dangerous’ identities that can undermine their contributions as parents and their potential to forge new paths in life. The risk framework has become an orthodoxy that shapes the work of social services and practitioners in the CJS. A prime concern of policy is to assess the potential of young offenders to reoffend and to pose a threat to their families. This orientation developed from the nineteenth century notion of the ‘dangerous classes’. Armed with the theory that danger can be predicted, governments authorised professionals to determine who was, or was not, ‘risky’ (Denney, 2005). While universal risk frameworks may fulfil an important function in helping to identify potential offenders and their support needs, and to protect against potentially harmful behaviour, a risk orientation may also be detrimental (McNeill and Weaver, 2010). Firstly, not all risks are predictable or preventable; secondly, in an effort to protect ‘us’ from ‘them’, increasingly incapacitating forms of control are sanctioned; and, thirdly, a focus on potential future offending punishes the offender for things they have not done and may never do. It also fails to recognise or nurture the potential of young fathers to make a positive contribution as parents and citizens.

An alternative, redemptionist approach takes as its starting point a compassionate understanding of human fallibility. It seeks to work with the fluidity of the life course and to forge non-criminal identities and life paths through re-orientations to past lives and future aspirations (Maruna, 2001; Laub and Sampson, 2003; Bottoms and Shapland, 2011; MacDonald *et al.*, 2011). The application of these ideas to young offender fathers has been pioneered by Meek (2007b, 2011). In her studies of possible future selves, she highlights the utility of supporting young men to develop their parental skills and identities while in custody. The liminal setting of prison can unravel the identities of inmates, but it also enables young men to withdraw from, and take stock of, their lives, and explore alternative pathways.

### Parenthood as a route to desistance

While desistance from crime should not be the only aim of supporting young offender fathers, it is a positive associated element. Re-offending is widespread among young offenders: the rate for eighteen to twenty-year-olds within two years of leaving prison is 67.9 per cent (Bottoms and Shapland, 2011; Ministry of Justice, 2015). However, as part of the process of ‘growing up’ most individuals do desist over time (Helyar-Cardwell, 2012). The factors that lead to persistence or desistance have been extensively researched. One strand of scholarship eschews static theories to explore the complex constellation of life course processes – individual, relational, spiritual, social, economic and structural – that influence the unfolding lives of offenders (Laub and Sampson, 2003; Farrall *et al.*, 2014). The economic and structural conditions that shape the livelihoods and citizenship of young people play a key role, as do local environments and peer group pressure (MacDonald *et al.*, 2011). It is also well established that family and intimate relationships are fundamentally important in these processes (Sherlock, 2004). Conventional values concerning family, home, children, employment and ‘doing good’ are strongly held by young male offenders; the high rate of re-offending noted above drops by up to six times if young people stay in touch with their families while in custody (Ditchfield, 1994). In this context, becoming a young father is a key transition that can create a new and positive identity to replace an offender reputation (Meek, 2011; Bottoms and Shapland, 2011). Parenthood becomes a key component of a ‘redemption script’: a constructed narrative that explains why offenders did what they did and why they have now put this behaviour behind them (Maruna, 2001).

The importance of social identity in these processes has been stressed by a number of researchers (Maruna, 2001; Meek, 2007b, 2011; McNeill and Weaver, 2010). Offenders with strong commitments to social goals, high levels of motivation and the confidence to plan for the future are less likely to re-offend (Maruna, 2001). A key debate in the literature concerns the extent to which concrete changes in behaviour are bound up with changes in identity and values. The direction of influence is difficult to disentangle, but both dimensions, identities and practices would seem to be inextricably linked (Helyar-Cardwell, 2012). The mechanisms by which such changes occur are also subject to debate. The notion of ‘turning points’ is commonly used in criminology research to pin-point the trigger for a concrete change in the direction of a life. However, these mechanisms are perhaps better understood as key moments, events or interactions that create changes in inner, biographical, dispositions. These may or may not lead to concrete changes in behaviour, or influence longer term trajectories. Such experiences may also accumulate in varied ways: as incremental nudges along a pathway, or as ‘eddies’ or ‘drifts’ in varied, sometimes random directions, subject to a host of intervening circumstances that make up a unique biography (Carlsson, 2012).

Given the varied factors that shape criminal journeys, Farrall and colleagues (2014) observe that unitary theories of desistance are less than helpful; the processes involved are complex and multi-faceted, and it is the intersection of these varied factors over time, and the salience of particular factors in individual cases, that need to be better understood by researchers and those working in the CJS (Farrall *et al.*, 2014).

### Professional support for young fathers

Professional support and interventions are also key factors in inculcating or reinforcing identities and life paths through parenthood and offending. Incarceration and a ‘punishment’ regime, grounded in a risk framework, run the risk of re-enforcing criminal identities, while support workers, so-called ‘normal smiths’ (for example, probation officers, Farrall *et al.*, 2014), use a redemption ethos as a route to desistance. Fatherhood programmes are thought to have been widely implemented in British young offender institutions over the past twenty years (Boswell and Wedge, 2002). Evaluations of such provision (Meek, 2007a; Buston *et al.*, 2012) indicate that they can be highly effective where they are flexible and tailored to the young men's unique developmental, rehabilitative and circumstantial needs. Without a central budget, however, there is no systematic or comprehensive provision. Instead, support is provided in piecemeal fashion by individual charities and ‘local champions’ of young fathers.

In the light of a dominant risk framework that may serve to marginalise young fathers, we set out here to understand the lived experiences of a small sample of young offender fathers, to explore the intersection of their parenting and offending identities and practices over time, to discern what helps or hinders and to consider what impact provision in the CJS has on their practices and values.

### The following young fathers study

The evidence presented here is drawn from a sub-sample of five young offender fathers and a sub-sample of practitioners working in the CJS in varied locations in the UK. They were interviewed as part of an ESRC funded study of young fatherhood (Neale and Davies, forthcoming). The study utilised Qualitative Longitudinal (QL) methods, taking the life course as the central organising principle and exploring changes and continuities in the lives of thirty-one young fathers who were followed over time. The qualitative framing enabled us to weave past and future into our data gathering and to gain insights into the lived experiences of young parenthood; in our view, subjective understandings are a crucial dimension of explanation (Bottoms and Shapland, 2011). While the sample is small, the accounts yield important insights in a context where there is currently no qualitative longitudinal evidence base on young offender fathers.

The five young men were from low-income, highly disadvantaged backgrounds, with fragile family and social ties. Two of the young men had been in foster care. All were school-age offenders, engaging in crime before the arrival of their first child and ranging in age from fourteen to twenty-two when they received their first custodial sentences. Jax, at the age of eighteen, was an expectant father while in custody, while Raymond (then also aged eighteen) went to prison shortly after the birth of his child. During the course of the study, two young men (Jason and Tarrell) had experienced further spells in prison. The fifth young man, Steven, was a longer term offender, having spent a total of seven years in custody.

Our analysis rests on both prospective and retrospective accounts of change. Four waves of interviews were conducted with Jason and Tarrell (2011–14) and two with Jax (2013–14). One-off life history interviews were conducted with Raymond and Steven (2014), who were recruited specifically for their custodial experiences. We had planned to track a sample of young offender fathers through their custodial sentences and into resettlement, generating insights into the unfolding of their lives in ‘real’ time, but we abandoned this idea. The site for our research, a Secure Training Centre, allowed interviews only on condition that the young men were supervised by front-line staff and the transcripts shared with the management team. In other words, access to the young men was governed by a ‘risk’ framework that would have compromised their confidentiality. It is important to note, also, that the prospective, longitudinal window afforded by this study is a modest one (two to four years). These trajectories are very much in the making and, as Laub and Sampson (2003) observe, it is only with hindsight over the longer term that we might discern more clearly the intersecting factors that shape these journeys through parenthood and offending.

## Findings

### Narratives of redemption

The majority of our longitudinal sample of young men described risk-taking, and, in some cases, criminal activity prior to the arrival of their first child. Becoming a father was commonly cited as a reason to modify such behaviour, a necessary part of the transition into a new identity and responsible adulthood. In this regard, early entry into fatherhood has positive connotations that run counter to its perception as a social ill (Neale and Davies, forthcoming). Professional mentoring for young fathers, received by approximately half of our full sample, was also seen as beneficial in preventing or modifying a drift into crime:

[W]ell if I didn't have our [son] like, I always say I’d either be doing proper bad drugs and stuff like that or I’d be in prison at least. Cause half of people who I used to hang around with, they are all in prison now. (Darren, aged 21, wave 1)

Tim [learning mentor] . . . he actually helped me a lot emotionally . . . well, say, if didn't meet Tim, I could be out robbing . . . selling drugs, doing whatever. Because I didn't have emotional sort of boundaries that – I couldn't care less about anything. So to get things off my chest with Tim, it helped me sort of stabilise myself and achieve what I can achieve reasonably. (Adam, aged 18, wave 4)

The redeeming power of young parenthood and critiques of the risk framework in the CJS were evident among the practitioners in our study:

A lot of offenders when they have a child will calm down and desist from offending because they have a different sort of, they have a different identity . . . They’ve gone from being whatever they saw themselves as before to being a responsible father. (Youth Worker: Young Offending Team)

There are some men who are dangerous and violent and shouldn't be involved in their children's lives. But they are a real minority . . . All that needs to be addressed . . . But . . . what tends to happen is young fathers get excluded, especially if they’re considered risky . . . It needs to be seen that they’re just as important as mothers. Someone needs to try and understand them and work with them. Yes, they might end up being too risky but then it's actually safer to identify who they are and work out what the issues are than just ignore them. (Staff Nurse, Health Education Lead, Secure Training Centre)

Among the young offender fathers, there was a strong drive to ‘do good’ as parents and citizens, and to learn from past mistakes. Their fatherhood status and family influences, particularly from their mothers and the mothers of their children, provided a strong counterbalance to peer group pressure:

Back then, I used to get in trouble. I was selling drugs and stuff . . . was just stood chilling with the wrong friends . . . trying to think that I was hard . . . And my mum started saying ‘when you gonna stop? . . . if you get locked up again rah rah rah’ . . . She’d say I’m the man of the house when my dad passed away . . . and I just thought . . . ‘I shouldn't be putting so much pressure on her’ . . . ‘let me go down the right path’ . . . And then turning my life around, with getting locked up . . . If I kept on going back to court, then . . . I could have got sent to jail. But with me having the kids, I think it changed the judge's minds . . . So, I tried to make my own path . . . elevated myself really . . . See, after me having my kids it opened up a better side to me . . . I were getting into like training schemes, football, college, and stuff like that. (Tarrell, aged 21, wave 1)

If it wasn't for [the secure training centre], I wouldn't be a father. I would not be the dad I am today . . . I felt worthless . . . But then again . . . you do the crime, you do the time . . . Like, my baby's mother, she was upset. And I explained to her, ‘I’m gonna come out as a better person.’ I did my part from in there . . . If I went back in there, I can literally . . . say I don't love my son [laughs]. Seriously, how if you’ve got your children out there, but you’re in and out of prison, how does that work? How does it physically work? It can't work! Me being a father should be a privilege. (Raymond, aged 20, life history interview)

One of the biggest challenges for me was, like, staying away from my mates . . . [my partner] would go mad [otherwise] . . . Because obviously I used to get in a lot of trouble . . . We used to take drugs and all that, and party . . . I got out of jail and, obviously, I thought, I can't do that anymore . . . I had to [stop] for myself and for [my daughter]. I didn't . . . want to go back to jail . . . I just wanted to stop, like altogether . . . stop being . . . a fucking idiot basically, do you know what I mean? See, there's a time to grow up, isn't there? (Jax, aged 19, wave 2)

These are life-changing narratives, redemption scripts that, for these young men, were wrought largely through the arrival of their children (Maruna, 2001; Helyar-Cardwell, 2012).

Paradoxically, while fatherhood is an incentive to desist, resorting to crime could become or remain a temptation where fathers have few resources to provide for their children. Selling drugs was a commonly reported activity, part of a widespread drug culture in depressed communities (MacDonald *et al.*, 2011). When, aged fifteen, Raymond found he was expecting a child, he started selling drugs for money, which led to a short custodial sentence. During the course of the study, Tarrell, on a downward path in terms of employment and training, and with relationship problems, went back to selling drugs; he spent over a year in custody for ‘possession with intent to supply’. Upon his release, he was supported to find work, and this concrete change was a route to maintaining a relationship with his children, and a strong rationale to desist:

Cause I’ll tell you now I probably . . . wouldn't have given, given a shit mate. I’d probably be just messing about now still. But . . . I think I might think about stuff more now. Cause I’m on . . . licence as well so I can't commit any crimes . . . I like being, I like having my kids, I like providing for them. And I’m in a better, better position now I’m working and such. (Tarrell, aged 25, wave 4)

### Re-offending

As Tarrell's case shows, while young fathers may be strongly motivated to replace a criminal identity with a parental one, such transitions do not happen instantaneously or in a linear direction. The fathers in this study described slippery, protracted journeys towards an ideal future self, a state of fragile desistance, in which the vigilance needed to maintain a new identity could sometimes falter (Laub and Sampson, 2003; MacDonald *et al.*, 2011). Relapses into offending behaviour, which the fathers described with regret, were most often attributed to relationship problems and difficulties in finding work, an adequate income, and a stable home. In such circumstances, the road to crime, or back to crime, continues to beckon, as existing theories of desistance clearly show (Maruna, 2001; Laub and Sampson, 2003; McNeill and Weaver, 2010). However, these were not necessarily pre-meditated journeys. A feature of impoverished lives is that time horizons shrink as people become preoccupied with day-to-day survival. Living in the moment means a reduced capacity to care for the past or plan for the future, circumstances that can engender risky behaviour. Jason's unfolding life reveals this pattern. Here we chart the complex factors that shaped his pathway through parenting and offending in early adulthood.

Jason's mother was a drug addict who died during his childhood. He had been close to her but not to his father, who was absent for much of the time. He spent part of his earlier years in the care system and had anger management problems, fuelled by alcohol, which led to his custodial sentences. At first interview, aged twenty-two, he had recently become a father within a fleeting relationship. Although not in a partnership, he was committed to being there for his son and supporting the mother:

I didn't want to be a dad ’cause for starters I’m unemployed . . . I’ve always wanted kids . . . in a . . . stable life and . . . it's far from stable . . . Work, having a nice home, doing healthy things, things that are good for your mind and that. And living in a council flat in a block of smack head flats in [a deprived area of the city] with no job wasn't ideal . . . Didn't even have a garden for him to play in . . . So I can't give him the best possible life. But . . . once he were born, it's crazy . . . nothing else matters. Everything you do is for him . . . It's impossible to describe, I think. It's just overwhelming. You are responsible for something that can't be independent and needs help . . . You have to be there for him, don't you . . . sacrifice things to make their life better. Like I used to . . . smoke weed. But I just stopped . . . I’ve got a crap dad so obviously I want to be total opposite and be a good example to him. I would never consider putting [son] in an ounce of life I’ve had . . . that's not a normal upbringing. (Jason, aged 22/25, wave1/4)

At first interview, Jason reflected back on his custodial sentence at the age of seventeen. He had stayed largely out of trouble for over three years and held on to the fact that his offences were in his past life, before he had children:

And I just . . . I’m so glad that I went to jail. I regretted it at the time like, but I’m glad now because it's just changed me. Not as much as being a father has changed me. But it's stopped me like fighting over silly things . . . and getting wrecked all the time . . . I used to think it was a coincidence I got arrested when I were drunk! . . . Obviously when you start thinking you realise . . . summat like 67 per cent re-offend and come back within a year. And when I was in there, I thought, ‘I’m not gonna be one of those 67 per cent’ and I’ve not been . . . If you are around people . . . who are committing crime, you take part don't you . . . if . . . not . . . [you’re] probably gonna get a lot further in life . . . But I get drunk . . . so that's not a good father because I’m blowing all that money . . . [and] when he gets older, it's not a good example to set . . . What I’ve done in past, before he were born, I can't alter that . . . [but] now I’ve got [my son] if someone came up to me throwing punches . . . I’d put my hand away . . . just say ‘get a grip’. ’Cause if I went to jail and I spent every day missing [my son], it's definitely not worth it now. (Jason, aged 22/23, wave 1/2)

A year later, following a ‘heat of the moment’ brawl in a pub, Jason received a second custodial sentence:

It were horrible not seeing my son . . . knowing I were missing the most important part of his life where he's . . . learning how to talk and walk . . . I just felt I’d let him down big time . . . I’ve let [the mother] down because she's had to cope with him for fifteen months . . . and I weren't supporting him financially . . . When I got out, we didn't have a bond. And, like, he’d come on visits, I’d pick him up and he’d want me to put him back down and it’d hurt me. And I’d say . . . to [mother of the child], ‘I don't want to see him for a bit.’ And she’d be like, ‘Why, you need to see him more often, not less.’ And I said, ‘it's hard, it breaks my heart . . . when I pick him up and he wants me to put him down and he runs to you’ . . . That's the price I paid for being an idiot. I . . . weren't someone important to him [in prison]. Where [as], now I am . . . we have a perfect bond . . . It just makes me realise that I should never commit a crime again . . . [not on] purpose . . . it's just taught me I’ll never go back [to prison] . . . Well I hope not . . . ’Cause I know I’ve still got stupid tendencies. I know how to get worked up far too easy. (Jason, aged 24, wave 3)

Jason was enjoying regular contact with his son at wave 3, but by the time of his final interview, a year later, life had taken a downward turn. When he began a new relationship, his son's mother blocked contact and sent an inflammatory text about his son's feelings for him that, in the heat of the moment, led Jason to retaliate. He was arrested for sending a text threatening to commit damage to property. Given his previous record of offences, this resulted in a restraining order that gave legal force to his loss of contact. Anguished over his relationship with his son, he was seeking to reinstate contact through the family court:

I’m gutted . . . ’cause I missed fifteen months of my son's life already. Well I’m missing more of it as the days go by. But if I’m in jail, I’m unable to fight for [contact] . . . He's three years old now . . . And the older he is, the more he's going to know, isn't he. I need to set a good example . . . I’m going through double the heartache [sighs]. [It’s] a whole lot more difficult because I’m missing him at different stages of his life . . . that I’ll never get back . . . It just hurts bad. (Jason, aged 25, wave 4)

**Figure 1.**
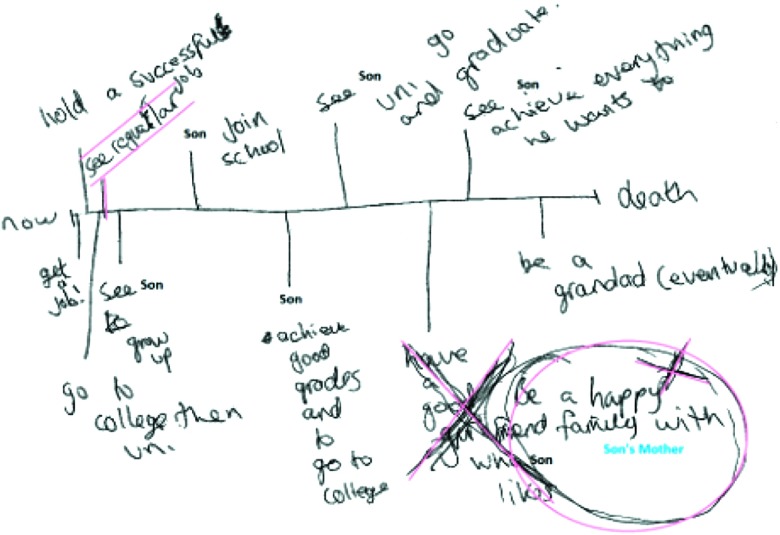
Jason’s future time map, revisited.

Over the course of the study, Jason's aspirations for the future did not waver. His time map, created at the age of twenty-two, shows how the arrival of his child created a sense of purpose in his life, built around conventional aspirations for parenthood, relationships, education and work. Revisiting the map at the age of twenty-five (see Figure 1), Jason was no longer seeking a new relationship, but wished to create a happy family unit with the mother of his child.

Over a four-year period, however, Jason had made little headway in achieving these aspirations. Nor, despite the best of intentions, had he managed to desist from crime. Whether he makes progress in years to come, and what factors will conspire to influence his journey, remains to be seen.

### Professional interventions and support

Professional interventions can make a significant impact on the journeys of young offender fathers, affecting their contact with children while in prison, their skills in becoming a parent and their life chances upon release. Maintaining contact while in custody is challenging for young fathers. As the custodial estate is shrinking, young offenders are increasingly placed further away from home, with an estimated 30 per cent held over fifty miles away (Summerfield, 2011). The children's mothers, who facilitate contact, may be unwilling to take their child into prison, and if they are under eighteen, they are classed as children themselves and must be accompanied by an adult. Added to these hurdles, visits per month are strictly limited and supervised. Extra visitation rights are granted for good behaviour, but CJS staff have to weigh up the risk factors. At the same time, the young men are confronted with bringing together two diametrically opposed and seemingly irreconcilable dimensions of their lives, their ‘good’ and ‘bad’ personas, in the same space and time. As Raymond explained, ‘It burnt me [not to see him] . . . but seeing him . . . would have drove me mad.’ In these circumstances, indirect contact by phone or letter was often preferred:

When you see people come in off visits, it hurts people more when they get visits, ’cause when the visitors are gone it's back to square one. Now me, I could just carry . . . I could wake up, make me phone call. As long as you get your phone call and see everyone's all right at home, that would make you feel good in the day. (Tarrell, aged 24, wave 3)

Indirect forms of contact are facilitated through *Storybook dads*, a voluntary sector course, running over several weeks and operating in over 100 UK prisons. Fathers learn to make and video record stories for their children, in the process developing communication skills and resources to play with their children. Successful completion of the course culminates in a special family visit where the storybook is presented to the child in an informal play setting, with low key surveillance. Tarrell, Steven and Jason had taken part in this scheme:

It's excellent being a dad, but not a dad on the inside ’cause you can't do much. I made my little girl a . . . CD storybook . . . It’d make me feel good . . . Like I’m still with them even though I’m not there. (Tarrell, aged 24, wave 3)

It were good to be honest . . . ‘cause [oldest daughter] has always had me there . . . I’ve got a really strong bond with her. It were . . . something where . . . I sorta kept familiar with her. She watches it every night. She loves it [laughs] to be honest. She's got it straight on repeat. It's quite good. (Steven, aged 26, life history interview)

Jason's experiences were less positive. Despite his best efforts, he was not granted security clearance for the family visit. He had attached importance to this visit, since he found it difficult to bond with his child under routine, strict surveillance in an adult prison:

I tried my hardest to get enhanced [visits] . . . and then the week before the visit, security came and said I weren't allowed to go . . . I’ve got security [risks] . . . I was fuming. I had to send that book out by post . . . The course didn't really teach no . . . father skills or anything like that . . . so I didn't achieve nothing from it . . . You have to be . . . proper trusted . . . and it takes months before you’re allowed . . . If you got to see [your child] . . . like in a playroom . . . with a nice camera in there, maybe an officer for trusted prisoners . . . that’d be good - ’cause you’re not bonding, sat on a seat with a table and a drink and a packet of crisps. How are you bonding? I did that for fifteen months . . . He's not looking forward to coming and sitting at a table for two hours. He gets bored and restless. Where, maybe, if we were . . . playing toys together and having a laugh, then maybe we would. (Jason, aged 24, wave 3)

In this case, the risk framework prevailed at the expense of a redemption ethos that would have facilitated Jason's relationship with this child.

### Fatherhood programmes

As indicated above, there is no systematic provision in the CJS that supports young offender fathers to develop their skills and confidence as parents. A generic, five week fatherhood course, *Fathers Inside*, is available for older fathers. Run by Safeground, a voluntary sector provider, and using group work, drama and games, it focuses on parental responsibilities, child development and skills to desist from crime. The course culminates in a performance during a special family visit. Steven found this valuable as it gave him a new perspective on his role as a father:

Everyone had different views which it were sort of good – to get a few views and ideas, you know . . . And different ways of putting it across. I did learn quite a lot, to be honest . . . Just your, your children's needs really. The needs that, you know . . . money can't buy . . . There's being there, being able to listen and a lot of that rather than going out and, yeah, you’ve got a brand new computer, new bike, new this, new that. But I’d prefer now to . . . not make as much money, but be able to spend more time with me kids . . . It's sorta . . . given me the drive to . . . better myself for them. Give them the things I didn't have. Spending time and, you know, having a good relationship with them. (Steven, aged 26, life history interview)

Few programmes exist that are specially tailored to the needs of young fathers and prospective fathers. A six-week course was developed in 2011 by a staff nurse in a Secure Training Centre. Called the Young Fatherhood Programme, it covers the practical elements of childcare, and the needs of children, with the young men tasked to care for an electronic (cyber) baby overnight. Developing an amicable working relationship with the mother of the child is a vital part of the programme, with input from a young mother. Beyond this, there is a broader focus on Sex and Relationship Education (SRE) and on life skills and personal development, with input from SRE practitioners and young ex-offenders. Of particular importance is the theme of personal development, of caring for oneself as an integral part of being a good parent. Drawing on the Good Lives Model (GLM) (Ward *et al.*, 2007), participants are encouraged to explore what they want and who they want to be in future:

When [there is] a pregnancy . . . you have a really quite powerful emotional response . . . They care a lot about it . . . What's interesting is most of them identify the same stuff. They want to have a good relationship. They want to have a job. They want to have somewhere to live . . . And so if they want those things, they need to work towards achieving those things. And it can be . . . really challenging. But it's basically about taking care of yourself in order to care for your child and have a good relationship with the child's mother . . . It's just to get them to . . . think about the fact that being a good parent, either now or in the future, is about developing your own life in a positive way. (Staff Nurse, Health Education Lead, STC)

It was this course that shaped Raymond's fatherhood identity and commitment:

The first fatherhood group, it was amazing. It literally taught me a lot of things . . . I know there's a lot of fathers inside. And there's a lot . . . that want to be involved, [but] are not involved . . . [or] don't want to be involved because they don't know how . . . ’cause there's no help out there [for dads]. (Raymond, aged 20, life history interview)

The holistic nature of this programme, the voluntary nature of the participation, and the redemptionist ethos that drives it are crucial to its effectiveness. While there is potential to roll out this provision across youth offending settings, it is currently limited to one institution. Only two young men in this study had benefitted from such support.

### Post-custodial support

If custodial settings are ideal for skills training and identity work, it is in resettlement that practical support can be offered to consolidate and mobilise a fledgling identity as a parent and citizen. The need for specialist input was acknowledged by practitioners:

I was dealing with . . . a lot of young fathers . . . I sort of made meself out to be a bit of a specialist just by, you know, professional qualifications before I became a probation officer.^2^ But . . . it would have been helpful to have a specialist from that field of work that could have done a . . . couple of sessions around parenting or a longer term bit of work. (Youth Worker, Young Offending Team)

The staff nurse at the Secure Training Centre had forged good links with community practitioners in order to create some continuity of care for her participants. Raymond was referred on to a local charity for young parents, where he was helped to find steady work. Jax and Tarrell received varied forms of practical support with their parenting. Jax, who was tagged within an Intensive Supervision Surveillance Unit, was allowed to stay with his newborn baby. Steven had seen changes in post custodial support over the years of his offending; since the age of fourteen, he had served five sentences, spanning seven years in prison. He talked of his crimes as a form of survival; with no home he used money from theft to pay for temporary accommodation. But he had recently received help to find a new home in a better locality:

[When] I come out of prison . . . I were just, sort of . . . doing me own thing. Trying to survive on me own . . . living however I could really . . . I’ve never found any help from probation or owt like that . . . It were just, ‘yeah you’re alright, go on, get yourself off’ . . . Apart from now . . . Probation is really good . . . supporting me to move, looking for another house which, which I’ve never really done before . . . It's supporting me with me court case to see me daughter and all that. Other day he pulled up at mine . . . just out of the blue. ‘Here Steven, I’ve done a bit of digging and that’, He's given me loads of information that he’d [printed off] in his own time . . . He's quite a good man. (Steven, aged 26, life history interview)

Jason, on the other hand, was not offered any specialist support as a parent upon his release. He was referred by probation to a VIAD course (for Violent Impulsive Angry Drinkers). Yet an intervention built around his ‘risky’ status was perhaps counterproductive. The participants were entrenched alcoholics in their middle years, some with severe mental health problems, whom Jason could not relate to. Overall, while post-custodial support can make a crucial difference to young fathers, provision is patchy and variable in terms of how far it is shaped by an ethos of redemption.

## Discussion and policy implications

Our findings show the significance that young offender fathers attach to their role and responsibilities as parents and their desire to ‘do good’ for their children. At the same time, the challenges faced by all young fathers are magnified for this group. They represent some of the most marginalised people in our society, living in varying degrees of poverty and deprivation that may go some way to explaining their chequered offending histories. In addition, their lives are shaped by their offender reputations and the risks they are presumed to present to their children, the mothers and society at large.

We have sought to document here the complex and varied ways in which the offending and parenting journeys of young fathers intersect over time. The transition to parenthood provides an acceptable identity and purpose to an offender's life, creating a strong incentive to ‘do good’ and desist from crime. At the same time, an offender identity and reputation can tarnish and constrain a fledgling identity as a parent and magnify the challenges faced by young fathers. Incarceration can reinforce these negative effects. Yet, with a redemptionist ethos in place, custodial settings can also be positive settings that foster the parenthood identities of young offenders. Regardless of these intersecting processes, however, it is clear that becoming a parent is not a ‘quick fix’ to desistance from crime.

Among the many factors that influence these processes, professional support that is built on an ethos of redemption would seem to be invaluable. This is all the more so when it is tailored to the needs of young fathers, extended to prospective young fathers, and delivered as early as possible in their offending and parenthood journeys. Our evidence suggests that this ethos is operating effectively within the CJS. Currently, it is not clear how widespread the new ethos is, or how quickly it may take hold among generic service providers, nor how best it might be incorporated into professional work where risk is the dominant focus. It seems to be driven in the main by ‘local champions’ for young fathers, practitioners residing in relatively isolated pockets of voluntary or privatised provision, who are pioneering new ways to develop their services, and are prepared to ‘go the extra mile’ to meet the needs of these young men. The practitioners represented here fall into this category. As wider evidence suggests, the crucial ingredient in the effectiveness of family support services and parenthood programmes is the quality of the relationships between practitioners and their clients (Rex, 1999; APPG, 2015: 11). This has implications for staff training and the time allotted to practitioners in their busy caseloads.

Organisational factors also play an important role. Well-established principles around co-ordinated, multi-agency provision, and continuity of care through robust referral systems, seem under-developed for young offender fathers (Dennison and Lyon, 2001; Helyar-Cardwell, 2012). Referrals that track young people from custodial settings through to their local communities, ensuring seamless support into resettlement, is an area ripe for development; another is the provision of holistic mentoring that can act as a conduit for referrals to other agencies. The way programmes are framed and how and when they are delivered is also crucial; it is important that they are pitched in ways that do not feel like additional punishment for young offender fathers because they are presumed to be ‘risky’. Provision such as the Young Fatherhood programme, which is voluntary and unconnected to court orders, is one step towards achieving this.

Returning to the theoretical framing of ideas about young offender fathers, our findings challenge the axiom that these young men are inherently ‘risky’. They suggest, instead, the utility of a dynamic, life course approach to criminal policy and practice that recognises the fluidity of life journeys, and brings ideas of redemption more centrally into the picture. The importance of a life course approach is now well established in the field of criminology. However, policy and practice settings are seemingly lacking a framework that engages with past journeys and future orientations, re-focuses on capabilities and resourcefulness, and enables ideas of redemption to be more fully articulated. Maruna (2001: 164) notes that re-biographing (clearing the slate on past criminal records after a number of years) is an established principle of the British legal system. Through the Sentencing Council, provision exists to take the parental status of young offenders into account. But young fathers go unrecognised (Helyar-Cardwell, 2012). They are sidelined here, as they are in other areas of provision (Neale and Davies, 2015). There is clearly more to be done to adopt and adapt a life course approach in custodial policy and practice. This would lead to wider understanding of the lived experiences of young offender fathers, and enable the provision of comprehensive, tailored support for these young men as they attempt to ‘do good’ in their lives.

## Notes

1 The percentage may be towards the upper end. Some young offenders (known as ghost fathers) hide their paternity if they are concerned they cannot make a financial contribution, or fear the potential removal of a child from the mother by social services (Kate Bulman, personal communication).

2 Since December 2014, support from the national probation service has been limited to only the top quartile of risk cases. The remainder are dealt with by community rehabilitation companies (Stephen Farrall, personal communication).
